# Analysis and comparison of algorithms for the tomographic reconstruction of curved fibres

**DOI:** 10.1080/10589759.2020.1774583

**Published:** 2020-06-29

**Authors:** Bernhard Fröhler, Tim Elberfeld, Torsten Möller, Hans-Christian Hege, Jan De Beenhouwer, Jan Sijbers, Johann Kastner, Christoph Heinzl

**Affiliations:** a Research Group Computed Tomography, University of Applied Sciences Upper Austria, Wels, Austria; b Data Science @ Uni Vienna, and Faculty of Computer Science, University of Vienna, Vienna, Austria;; c Imec-Vision Lab, University of Antwerp, Antwerp, Belgium; d Research Group Visual Data Analysis, Zuse Institute Berlin, Berlin, Germany

**Keywords:** Fibre-reinforced polymers, fibre reconstruction, curved fibres, fibre visualisation

## Abstract

We present visual methods for the analysis and comparison of the results of curved fibre reconstruction algorithms, i.e., of algorithms extracting characteristics of curved fibres from X-ray computed tomography scans. In this work, we extend previous methods for the analysis and comparison of results of different fibre reconstruction algorithms or parametrisations to the analysis of curved fibres. We propose fibre dissimilarity measures for such curved fibres and apply these to compare multiple results to a specified reference. We further propose visualisation methods to analyse differences between multiple results quantitatively and qualitatively. In two case studies, we show that the presented methods provide valuable insights for advancing and parametrising fibre reconstruction algorithms, and support in improving their results in characterising curved fibres.

## 1. Introduction

The usage of fibre-reinforced composites has seen a strong increase in industry over the last decade. This is especially true for sectors such as aeronautics or automotive, where the combination of light weight with high strength and durability is desirable [1,2]. In such materials, properties such as resilience to stresses in a specific direction are determined by the characteristics and distribution of the fibres, such as their length, orientation or location. For understanding the properties of a specific material, it is therefore vital to be able to analyse all relevant characteristics and distributions. X-ray computed tomography (XCT) as a non-destructive testing technique [3] is considered the most suitable technology for acquiring high-resolution scans of such materials [4]. In the step following the XCT imaging, algorithms or data processing pipelines extracting characteristics of single fibres from the computed tomography scans are required. A number of methods have been proposed in literature, which we call *fibre reconstruction algorithms*.

In the work by Salaberger et al. [5,6], e.g., a pipeline is applied with successive volume processing steps such as Gaussian smoothing and binary thinning to segment the centre lines of each fibre. Glöckner et al. propose a model-based approach using Monte-Carlo techniques for the extraction of characteristics of single fibres [7]. Konopczynski et al. [8] show that deep neural networks can be used for detecting fibres as well. Elberfeld et al. optimise the characteristics for each fibre by iteratively reconstructing directly to a fibre model [9]. Methods increasingly also start to get released in source code, such as the Insegt Fibre approach by Emerson et al. [10], enabling better reproducibility.

To analyse the extracted characteristics of fibres in single results of fibre reconstruction algorithms, tools such as FiberScout [11] can be used. These tools enable users to answer questions such as where in the dataset fibres are oriented in a specific direction, or where particularly short or long fibres are located. Often, also several fibre reconstruction algorithms, different parametrisations of such algorithms, or multiple computed tomography scans of the same specimen with different scanning parameters need to be compared. In such situations, the tool FIAKER [12] enables users to compare and analyse multiple results at once. Both tools aim at the analysis of samples with straight fibres. In FIAKER, fibres are even modelled and visualised as cylinders, thus it can not be used for curved fibres.

In many application cases, however, the analysed fibres *are* curved [13]. Especially in recent years, a multitude of experiments with various fibre materials have been conducted. For example, natural fibres are used in an effort to make fibre-reinforced materials more environmentally friendly [14]. In additive manufacturing of fibre reinforced parts, continuous fibres are used [15]. Advanced approaches for manufacturing of fibre reinforced parts, e.g., infuse resin into fibre fabrics [16]. In all of these examples, fibres are curved, either because they consist of a non-stiff material and thus bend easily, or they are very long, so that modelling them as perfectly straight does not match reality closely enough. Users analysing curved fibres are facing at least two of the challenges laid out by Heinzl and Stappen [17]; the *Integrated Visual Analysis Challenge*, as the data is complex enough to make the analysis with general visualisation tools exceedingly hard; as well as the *Visual Debugger Challenge*, as without specialised tools it is impossible to optimise fibre reconstruction algorithms for curved fibres.

In this work, we therefore extend the methods implemented in FIAKER [12] by algorithms that can deal with curved fibres. The proposed methods can be used on the one hand by developers of fibre reconstruction algorithms, in order to improve the algorithm itself, e.g., to identify problems such as fibres in a reference dataset without a matching fibre in any of the results. On the other hand, they can be employed by users who work with such algorithms and want to find the most suitable set of parameters for a given algorithm when determining fibre characteristics for a specific material system. Our contribution in this work therefore includes the following:

• the definition and evaluation of fibre dissimilarity measures for curved fibres,

• methods for visualising and comparing multiple representations of the same specimen containing curved fibres, extending the FIAKER tool,

• and two case studies applying these methods on synthetic and real-world computed tomography scans of specimen containing curved fibres.

## 2. Methods

**Figure 1.**
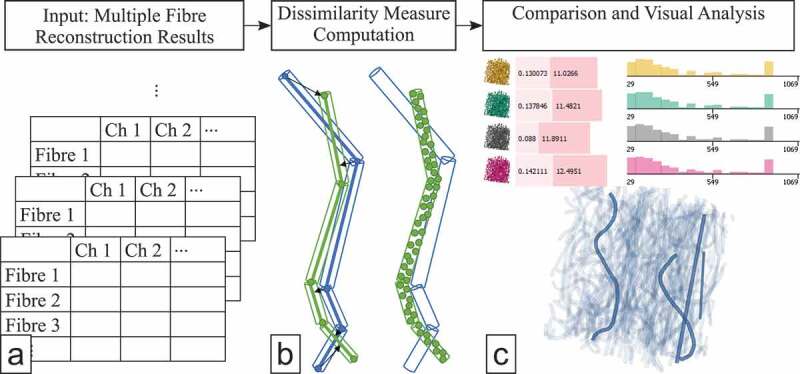
The workflow of FIAKER starts with multiple precomputed fibre reconstruction results to be analysed and explored (a). The dissimilarity computation to a user-defined reference (b) is incorporated into the proposed visual analysis methods (c).

A typical analysis workflow involving FIAKER is shown in Figure 1. FIAKER can be employed to compare different fibre reconstruction algorithms, varying parametrisations of these algorithms, or it can be applied to the analysis of reconstructions resulting from varying imaging settings. In any of these cases, the analysis workflow starts where multiple results from fibre reconstruction algorithms are available. If not otherwise specified, the term *result* will be used to refer to a single outcome of a fibre reconstruction algorithm. Extending the methods in FIAKER [12] to our purposes involves finding a suitable way to model the curved fibres and to visually represent them, as well as devising a dissimilarity measure to find matching curved fibres in the various results.

### 2.1. Curved fibre representation

For the numeric representation of curved fibres we employ a number of fibre points along their central lines, and their respective diameters at each point. With this information, fibre segments can be constructed in the form of cylinders. The points along the central line can be placed either in constant distance from each other, or adapted to the local curvature of the fibre, i.e., more points are stored in areas where the fibre features a higher curvature. The final curved fibre is thus represented as a number of segments of cylinders along the fibre’s central line.

### 2.2. Dissimilarity measures for curved fibres

To determine the best-matching fibre in the reference for each fibre in a result, we require a dissimilarity measure capable of dealing with curved fibres. For this purpose, we extended both distance-based and overlap-based measures from the original FIAKER tool to make them usable for curved fibres.

**Figure 2.**
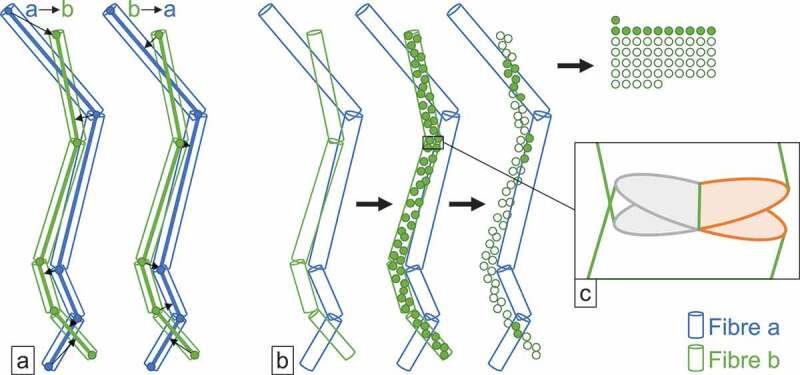
Principle behind distance-based (a) and overlap-based (b) dissimilarity measures; the zoom-in shows regions of potential error for the overlap measure (c).

#### 2.2.1. Distance-based measures

Analogously to measuring similarities between fibre tracts [18], we provide a distance-based measure based on the distances between points and line segments. The dissimilarity is computed as the average over the shortest distance of all fibre points of fibre a to the closest part of fibre b. More formally, when a curved fibre a is given as a sequence of line segments, specified by a list of m points along the fibres’ central line $p_{a}=p_{a}^{1},p_{a}^{2},\ldots ,p_{a}^{m}$, then our distance-based dissimilarity between two fibres a and b can be written as

(1) $$d_{a,b}=1/m\text{\textbackslash{}overm}\sum_{k=1}^{m}dist(p_{a}^{k},p_{b})$$

where $dist(x,p_{y})$ returns the shortest Euclidean distance between point x and the line segments specified by the points in $p_{y}$. This measure is directional, i.e., $d_{a,b}$ will be different from $d_{b,a}$. We therefore compute the measure in both directions, as shown in Figure 2a and take the minimum of the two values as final dissimilarity.

#### 2.2.2. Overlap-based measures

The principle for overlap-based measures for curved fibres is very similar as respective measures for straight fibres: We sample a fixed number of points on one fibre and check whether these points are contained in the fibre to be compared. In the sampling step, we assign each cylinder segment a number of sampled points, according to the ratio of the segment’s volume versus the total volume of the fibre. This way we guarantee that the sampled points are equally distributed among all fibre segments, and we do not sample more densely in short fibre segments as compared to long fibre segments. Figure 2b visualises this process: it shows a regular distribution of the sampled points over the whole fibre. The overlap dissimilarity is computed as the number of sample points contained in the other fibre, divided by the total number of sampled points. For the final dissimilarity, we multiply the outcome by the ratio of the volumes of the two fibres, such that a small fibre completely contained in a larger fibre does not get assigned a value of 1, which we want to reserve for the case where two fibres match exactly. We refer to this overlap dissimilarity weighted by the volume ratio as $do_{3}$ throughout the rest of the paper. This overlap measure does not consider potential overlap or openings of straight cylinders with orthogonal caps in the region where two segments meet, as depicted in the zoom-in of Figure 2c: The space on the left between the two regions marked in grey is covered by the cylinders of both fibre segments, while the gap on the right between the two regions marked in orange is not covered by any of the fibre segments. Therefore, the outcome can slightly deviate from the true overlap of two curved fibres. For our purposes, the accuracy is sufficient, though, since our main interest is not so much on highest precision regarding overlap, but rather on the correct relative ranking of the dissimilarities. In this regard, we found the overlap-based measure as defined above suitable for all our purposes. For the containment check of the sampled points in the other fibre, we can check the cylinder containment, i.e., whether a sampled point is somewhere between the two planes containing the upper and lower disk of the cylinder, and it is not more off from the central line than the cylinder radius. For our curved fibres, we need to perform this check against each fibre segment. This means that when computing this measure to determine best matches between two results, the computation effort increases quadratically not only with the number of fibres but also with the number of fibre segments.

The matches determined by the overlap-based measure follow most closely what domain experts would expect of which fibres match. Its calculation, however, requires more computational effort than distance-based measures. When the number of results to be compared or the amount of fibres analysed becomes larger, we can apply the same optimisation as applied in FIAKER for straight fibres: When comparing two results A, B, we first compute the distance-based measure between all possible fibre pairs a,b where a∈A and b∈B. Then, we rank the pairs by ascending dissimilarity, and only compute the overlap-based measure for the first S pairs as determined by the distance-based measure. While the first best match with the distance-based measure often might not be the best match according to the overlap-based measure, it will be among the first few. Thus, setting S lower will increase the performance while increasing the chance of missing the actual best match. For our experiments, setting S to 25 was determined to result in the best match being found in all investigated cases; but if needed it can be adapted according to user requirements.

### 2.3. Visual analysis

Before going into the details of the extensions applied to FIAKER to make it suitable for curved fibres, we will first give a short overview over the analysis methods and its capabilities.

#### 2.3.1. FIAKER

**Figure 3.**
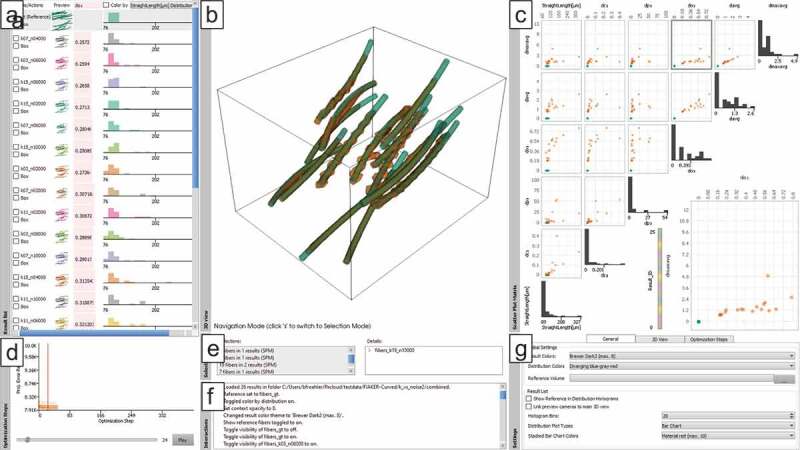
FIAKER analysing a list of 26 results (a) of fibre reconstruction algorithms aware of curved fibres, two of which are shown in the 3D view (b). A scatter plot matrix (c) provides an overview over the properties of all fibres; a step chart (d) enables inspecting single results of the optimisation step; selection (e) and interaction (f) protocols keep track of analysis steps, and finally a settings view (g) enables fine-grained control of the visualisations.

The main interface of FIAKER with the new extensions and a collection of results with curved fibres loaded is shown in Figure 3. In a result list (see Figure 3a), the name, a preview, a stacked vertical bar chart showing configurable measures as well as a distribution chart are shown for each analysed result. The preview shows a miniaturised view of the whole dataset, fibres in it as well as the distribution chart are assigned a characteristic colour for the specific result. This colour is used also in the larger 3D view and the scatter plot matrix, so that it can be easily discerned across all views to which result a fibre belongs. For each result, an average dissimilarity score to the user-defined reference is computed. It is displayed in the stacked vertical bar chart and can be used for a first quick quantitative insight into the performance of a specific result.

A detail view (see Figure 3b) provides a closer spatial look on selected results. This view can be used to select fibres at a specific location through drawing a selection rectangle or through clicking on a single fibre. Selections are propagated also to the scatter plot matrix and optimisation step charts. Selected fibres are shown with high opacity, and non-selected fibres are shown with low opacity for context information. Optionally, the diameter of non-selected fibres can also be reduced by a user-defined factor. Fibres can be coloured either by the result for identification, or by the characteristics distribution selected in the result list.

The scatter plot matrix (see Figure 3c) enables a detailed inspection of the fibre characteristics. Each dot represents a single fibre. Fibres are always coloured by the same scheme as fibres in the detail view. Here, fibres can be selected by their characteristic values; when selected they are highlighted in black.

Additional views optionally show details on the current selection (see Figure 3e), where the user can also refine his selection. Another view lists past interactions (see Figure 3d) for a better overview over the analysis history, and through a dock-able settings view (see Figure 3f), multiple display options can be adapted to the current analysis needs. For more details please refer to the original publication about FIAKER [12].

#### 2.3.2. Curved fibre visualisation

For visualisation purposes, we require the fibres to be available in the fibre points representation as described in Sec. 2.1. We create line segments between every consecutive pair of points on the central line of the fibre. In order to create a smooth appearance of the fibre, we apply a tube filter around the central line, which smooths discontinuities at points where the curvature or the orientation of the fibre changes. An example of this visualisation can be seen in Figure 3b. To provide more detail on the exact recognised shape of the fibre, FIAKER also provides the central line alone as optional alternative visualisation. This way of displaying curved fibres significantly increases the computational effort compared to the visualisation of straight fibres. Every single fibre segment requires as many surface primitives as a full curved fibre does. One typically targets a highly accurate representation with many, e.g., more than 100 fibre segments. Simultaneously, also often a high number of fibres (e.g., more than 10.000) are analysed. In the current version of our implementation, the user can change two parameters to adjust the rendering speed to his or her needs. First, one can reduce n, the number of faces used for the fibre segment surfaces, which defaults to a value of 12. It can be reduced down to a minimum value of 3, which reduces the surface polygons to be rendered by one quarter. It also results in a triangular outline when cutting through the fibre, though. Additionally, the parameter s, the step size over the segments, can be tuned to regulate the accuracy of curvature. The default value of 1 for s means that a line segment is created for every pair of consecutive fibre points. Integer values higher than 1 result in s−1 fibre points being skipped in the creation of segments.

### 2.4. Implementation details and performance

The methods described in this work were integrated directly into the FIAKER tool, which is implemented as a module of the open_iA framework [19]. open_iA is written in C++ and makes use of the Qt, VTK, and ITK frameworks. It is available as open source on GitHub.^1^

The case studies we present in the next section only contain up to approximately 250 fibres. This is quite small when compared to typical real-world datasets, which can contain up to a few hundred thousands fibres. Some performance details are provided here to give an idea about how well our analysis methods would perform on larger datasets. Because the calculation of the dissimilarity measures requires the most computational effort we focus on this part here. We here report the performance for the two ensembles analysed in the case study below. The computations were performed on a Xeon E5-1660 v4 with 64 GB of RAM. For the ensemble with 40 results, each result with 215 fibres on average and each of these with an average of 70 segments, the unoptimised computation of the overlap measure took 37 minutes 36 seconds (while for the smaller ensemble with 25 results, 16–18 fibres each and an average of 50 segments, it took approximately 5 seconds). Note that all computation times given here are single-core computation times. The computation was parallelised using OpenMP, and since it is very well parallelisable, the actual real-time computation time was only approximately 3 minutes (less than a second for the small ensemble). In contrast to the overlap measure, the distance-based measure only took 20 seconds (140 milliseconds) to compute. When the distance-based measure was used as an optimisation base for the overlap measure, as mentioned in Sec. 2.2.2, computation time was reduced to 4 minutes 20 seconds, with an overall real-time execution time of 30 seconds. For the small ensemble, this optimisation is not useful: In its current form, it is set to compute the overlap-based measure for the 25 best-matching reference fibres according to the optimisation base. In the small ensemble, however, there are only 16 fibres in the reference, and the unoptimised computation is already fast enough. The number of required comparisons for the optimisation base still increases quadratically with the number of results, the number of fibres, as well as the number of segments in each fibre. The calculation in its current form would therefore take approximately 13 days (on a single core) for a hypothetical ensemble of 40 results with 100.000 fibres each, and 20 segments per fibre. For the occasional analysis, this might be acceptable, since the computation only has to be performed once and is cached for the subsequent analysis steps. For a more frequent analysis of ensembles with such large result, new optimisation strategies, for example based on spatial subdivision schemes, would have to be devised. So far, however, for our purpose of doing a parameter space analysis, it was beneficial to focus on one or more smaller regions, as it is much easier to manually verify the reference for such a smaller region.

## 3. Results and discussion

We evaluate our extensions of FIAKER to curved fibres on two datasets: A synthetic dataset used in exploring a new fibre reconstruction algorithm, and a parameter study of an existing algorithm on a real PET fibre dataset.

### 3.1. Synthetic dataset

**Figure 4.**
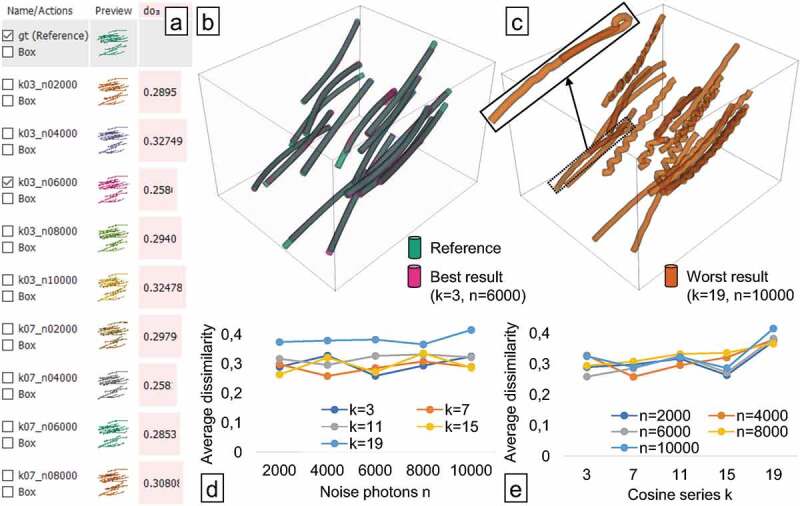
Analysing fibre characterisation results from a synthetic dataset analysed in FIAKER: inspecting the list of results (a) and a comparison between reference and best result (b). The worst result exhibits many fibres curved back onto themselves and with wriggly shapes (c). Average dissimilarity to reference plotted over the parameter ranges of noise photons (d) and cosine series k (e) parameters.

This small, synthetically created dataset is modelled after short glass fibre reinforced polymers. The ground truth with 16 slightly curved fibres is visualised in green in Figure 4b. The goal of this case study was to explore a fibre extraction algorithm in an early development stage, which employs an optimisation based on a cosine series representation of the fibres. This is an extension to the algorithm presented by Elberfeld et al. [20], which employs a piecewise linear representation of curved fibres. More specifically, we investigated the influence of two parameters of this fibre extraction algorithm on the result, namely n, the number of noise photons employed in the projection phase of the algorithm, and k, the number of combined functions in the cosine series, i.e. the number of weights in the cosine series representation. We chose five different values for k in the interval 3–19 with an increment of 4, and 5 values for n in the interval 2000–10,000 with an increment of 2000. We then created 25 results by running the algorithm with every possible pairing of these values.

The generated results contain 16–18 fibres. For each fibre, in each of these results the dissimilarity to the reference is computed. In Figure 4a, the average dissimilarity for the whole result based on overlap ($do_{3}$) is selected to be shown as horizontal, light-red bar chart in the middle of the result list. This enables to choose the ‘best’ result, i.e., the result with the smallest average dissimilarity, as shown in Figure 4b as overlay (magenta) together with the ground truth (cyan), which was created with k=3 and n=6000. However, k=7 and n=4000 resulted in approximately the same average dissimilarity. The ‘worst’ result (orange), i.e., the result with the highest average dissimilarity, shown in Figure 4c, was produced when choosing k=19,n=10000. In Figure 4d, the average dissimilarity is plotted over the noise photons. There is no clear trend visible, but four of the curves (one for each k value) have their minimum between 2000 and 6000. This is counter-intuitive, as more photons should lead to a better quality as there is less noise in the projections. In the fibre reconstruction algorithm, the number of metric computations is limited. A reason for this behaviour could be that we are seeing the effects of a trade-off between noise photons and number of optimisation iterations, which features a sweet spot for the noise photons at around 4000. As the noise photons are sampled randomly, it might also be necessary to compute an average over multiple results with the same amount of photons to get more conclusive results. In Figure 4e the average dissimilarity is plotted over the parameter k. Here, a clearer trend can be observed – higher values of k tend to show higher dissimilarity. Figure 4c clearly shows the reason for this trend – higher values of k lead to a wriggly appearance of the fibres.

The overlap measure cannot detect automatically if a fibre loops back onto itself. This is however easy to spot in the polygonal surface visualisation shown in FIAKER, as, e.g, shown in the zoom-in in Figure 4c. In the volume rendering utilised by the algorithm developer, this could not be recognised. This seems to be a common occurrence when utilising the cosine series representation, and there is no easy way to penalise such behaviour. Also, even for the best result, it can be seen that fibres tend to be slightly shorter than the according reference fibre. It might therefore be that the cosine series is not the best representation to be used in this kind of optimising fibre reconstruction algorithm. These insights prove that FIAKER provides valuable insights into the design of a fibre reconstruction algorithm being currently in development.

### 3.2. Strongly curved PET fibres

**Figure 5.**
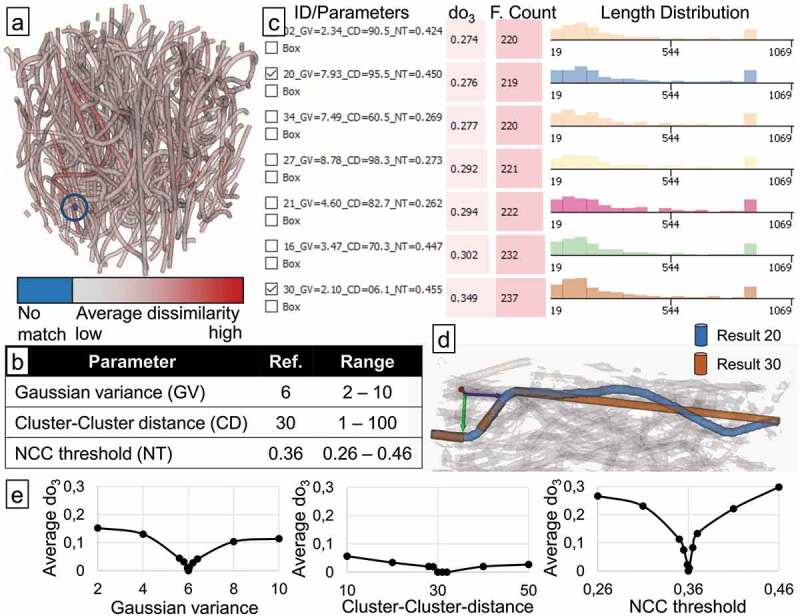
Parameter study of template matching based fibre reconstruction algorithm applied to strongly curved PET fibres in PP. The average dissimilarity to the best match on the other results is mapped on the reference result (a) as an overview over the variance in the ensemble. 40 results were generated, varying three parameters (b), listed here are seven results with highest dissimilarity (c). Low Gaussian variance (GV) and low Cluster-Cluster distance (CD) result in fibres being broken into multiple parts (d). A sensitivity analysis around the reference shows the NCC threshold parameter (NT) to have the highest influence on results as measured by $do_{3}$ (e).

We evaluated the proposed methods also on the analysis of a dataset with short, strongly curved polyethylene terephthalate (PET) fibres in polypropylene (PP). The goal here was to perform a parameter space analysis of a fibre reconstruction algorithm based on template matching. The reference dataset was created with the same algorithm by hand-tuning the parameters and is shown in Figure 5a. Three parameters were varied: the *Gaussian variance*, the *Cluster-cluster distance*, and the *NCC threshold*; for a detailed description of these parameters and typical values for them, we refer the interested reader to the PhD thesis by Salaberger [13]. We created 40 parameter sets using Latin hypercube sampling in intervals around the ideal parameter settings used for the reference, which are summarised in Figure 5b. In the reference, the algorithm identifies and characterises 214 fibres. Over the complete ensemble of results, the number of fibres varied from 203 to 237.

In Figure 5a, the reference result is shown, where each fibre is coloured according to the average dissimilarity to the best match among all the results. One very short fibre close to the edge is coloured in blue and highlighted with a blue circle for better visibility in the figure. This fibre had no match in the other results, closer inspection shows that the algorithm broke down what should be a single fibre into five separate fibres. This happens close to the border of the analysed region of interest, and we also see that several other extremely short fibres are identified close to the edges. So for the final length distribution output, the region at the border should be ignored.

To analyse the sensitivity of the results to changes in any of the three parameters, we sampled additional results centred on the reference, where we only varied one of the three parameters, while leaving the other two at the values of the reference. For the Gaussian variance and the NCC threshold, the same range was used as in the ensemble with 40 results. For the Cluster-Cluster distance, a different range of ±20 around the reference value had to be chosen, since the original range of 1−100 did not have the same range from the reference in both directions. We split the sampled region into 200 equally spaced parts and placed samples at 1,5,10,50, and 100 parts away from the centre in both directions. This yielded 10 results (5 per side) for each of the three parameters. For each of these results, we computed the average dissimilarity $do_{3}$ to the reference. Plotting $do_{3}$ over the parameters, as shown in Figure 5e, clearly shows that changing the NCC threshold by far has the highest influence on the result, with an average dissimilarity of up to 0.3.

When sorting the results by their average overlap dissimilarity to the reference, and inspecting the results with the highest dissimilarity as shown in Figure 5c, it is also apparent that the *NCC threshold* parameter influences the result most: All of these seven results with the highest average dissimilarity to the reference, out of the total of 40 results, are close to the upper (e.g. 0.455, 0.447) or lower (e.g. 0.262, 0.273) end of the sampled range for the NCC threshold parameter. For analysing the influence of the other parameters, we compared result 20 and 30, which differ mainly in the Cluster-Cluster distance parameter, but also in their Gaussian variance. From a visual inspection of these results, we identified a fibre in result 20, where the same fibre is broken down into three parts in result 30, as shown in Figure 5d. Due to little smoothing in result 30, the fibre is split up into three parts, exactly in the spots where there is high curvature in the fibre. The following analysis of whether parts belong to the same fibre is affected by the Cluster-Cluster distance parameter; the low value in result 30, combined with the high angle difference between the three parts, leads to these parts not being merged. In contrast, in result 20, more smoothing is applied due to the higher Gaussian variance. Additionally, the Cluster-Cluster distance parameter is larger, increasing the chance of merging disparate parts. We can conclude that the combination of little smoothing with a small NCC threshold leads to fibres being broken up into multiple parts. We can see this also reflected in the Fibre Count (F.Count) of result 30 in Figure 5c, which is the highest of all results.

A further insight from the fibres in Figure 5d stems from the seeming inconsistency of the perfectly straight fibre in result 30 with the corresponding part of the matching fibre in result 20, which even in this area is not fully straight, but bends upwards and downwards. The central line (not shown here) for the long, straight part of the broken fibre in result 30 was segmented very similarly to that part of the curved fibre in result 20. Only when writing the results, the algorithm decided that the fibre was not curved enough to store as multiple fibre segments. It therefore only stored start and end points and marked it as not curved. This might be suitable for situations where some fibres are strongly curved and others only very little. In the analysis scenario here, it could be misleading in further analysis. It can be deduced that the criteria for the decision of whether a fibre should be stored as curved requires better fine-tuning to the specific analysis scenario.

## 4. Conclusion and future work

We increased the applicability of the FIAKER tool [12] by extending it to curved fibres; it can now be used in a much wider range of application areas. In contrast to previous analysis with FIAKER, where only ensembles of up to eight results were analysed, in this work we put the focus on parameter studies with ensembles of up to 40 results. In the case studies presented in this work, we could show that the proposed method supports developers in the design of fibre reconstruction algorithms and pipelines. More specifically, we showed that the optimisation based on a cosine series representation is not ideal for curved fibres, if there is no way of penalising self-intersection during optimisation. We also investigated the influence of parameters on the fibre reconstruction of a PET fibre dataset. We conclusively determined the parameter with the highest influence on the results, showed what effect specific parameter combinations have on the results, and uncovered the importance of fine-tuning the criteria for when to consider a fibre as curved to the specific analysis scenario.

One potential to be exploited for future work is the exploration of other representations for curved fibres for optimisation purposes as well as ways of introducing penalty terms in said optimisation. We are also looking forward to do further evaluation on other types of materials, such as long glass fibre-reinforced polymers or carbon fibre reinforced polymers, or even on textile fibre fabrics.

During the analysis, it turned out that the methods proposed in this paper might not scale well to the analysis of a much larger number of results. So additional methods suitable for such larger ensembles might be a topic of future work as well.

## Note

1. https://github.com/3dct/open_iA
